# Simple rules and self-organisation: A complex systems’ perspective on South Africa’s COVID-19 response

**DOI:** 10.4102/jamba.v13i1.1013

**Published:** 2021-05-13

**Authors:** Christo Coetzee

**Affiliations:** 1 School for Geo-Spatial Science, Faculty of Natural Science, North-West University, Potchefstroom, South Africa

Since late December 2019, the world has been in the grip of the coronavirus disease 2019 (COVID-19) pandemic (Peng, Ho & Hota 2020:1). Originating in Wuhan, China, the disease spread rapidly across the globe because of the interlinked nature of the global economic and transport networks (Wolff 2020:2). Specifically, Imai et al. (2020:1) reported that by as early as 22 January 2020, a total of seven cases had been reported in countries, including Thailand, Japan, South Korea, Taiwan and the United States. These first infections outside of mainland China were only the precursor for the devastation to follow, as the exponential increase in infections overwhelmed many healthcare systems across the globe causing shortages in Intensive Care Unit (ICU) facilities, personal protective equipment (PPE), sanitation equipment and ventilators (Anderson et al. 2020:932). These medical shortages combined with symptoms of the virus impacted many developed and developing countries with substantial loss of life reported. The significant increases in infection and mortality rates prompted governments to take preventative actions to ensure the safety of their citizens. The government of South Africa was one such state and on the 15th of March 2020, the country’s President Cyril Ramaphosa declared a national state of disaster in terms of the *National Disaster Management Act* (57 of 2002) as a response to the impeding COVID-19 outbreak in the country. The declaration of the national state of disaster was a necessary step that needed to be taken by government to ensure the streamlining of governments human and financial resources and to allow sector specific emergency legislation and regulations to be formulated to tackle the outbreak of COVID-19 head on. The most prominent regulation put in place by government was the declaration of a nationwide ‘lockdown’ that intended to limit human and economic movement for an initial 21 days (26 March 2020 – 16 April 2020) (Costa & Tumagole 2020:3). As part of this initial lockdown, some basic regulations such as prohibition of gatherings of more than a 100 people, social distancing protocols, closure of schools and closure of businesses rendering non-essential services were put in place (Jaja et al. 2020:1077). It was believed that these initial lockdown regulations were necessary to implement as a way to flatten the curve of infections whilst providing hospitals with the opportunity to augment their ICU facilities and to bring in additional ventilators, PPE and sanitation equipment to cope with the expected surge in infected patients. Once the initial lockdown period lapsed on the 16th of April, the government reviewed data pertaining to infections and mortalities associated with COVID-19 in the country and took the step to extend the lockdown for an additional 2 weeks up and till the end of April. At the end of April, government did not end the nationwide lockdown but instead started to implement a risk-adjusted approach where sectors of the economy and society would systematically be reopened. As such, during the month of May, South Africa moved from level 5 lockdown or a ‘Hard lockdown’ to level 4 and then to a level 3 lockdown that allowed for the reopening of some sectors of the economy and social services such as opening of schools.

The initial move towards lockdown was well received by South African citizens, with 77% of South Africans participating in online poling at the time of strongly supporting the move (City Press 2020). However, with the passing of time and the formulation of more and more regulations to realise governments risk-based approach, the support for lockdown has waned significantly, with only 38% of participants in online poling still showing strong support for lockdown at the beginning of May (City Press 2020). Arguably, the formulation of additional laws and regulation to regulate various sectors of the economy and society seems to have played a strong role in eroding people’s support of the initial lockdown, with often contradictory or even irrational regulations being released by government departments. The extent of this problem came to fruition on the 2nd of June 2020, when Judge Denis Davis in the case of *De Beer, Liberty Fighters Network and Hola Bon Renaissance Foundation versus The Minister of Cooperative Governance and Traditional Affairs* (case 21542/2020) in the Gauteng High Court ruled that the regulations promulgated by the Minister of Cooperative Governance and Traditional Affairs with respect to alert levels 4 and 3 were not only in some instance irrational and contradictory but also crucially could encroach and limit citizens constitutional rights through their implementation. Judge Davis ordered the state and the Minister of Cooperative Governance and Traditional Affairs, to within 14 of the court order, review existing regulations and amend them in such a way that they no longer infringe on citizen constitutional rights. However, in this 14-day period, the existing level 3 regulations would remain in place. On the 3rd of June, cabinet signalled their intention to launch an urgent appeal of the High Court Judgement to the Supreme Court of Appeal. The Supreme Court of Appeal heard the case on the 24th of June 2020 and reserved the judgement in the matter. The waning public support and legal complications faced by the South African government beg the question, how could government implement an appropriate response to the threat of COVID-19 in South Africa differently? The answer to this could lie in some of the basic principles of complex adaptive systems theory, that is, simple rules and self-organisation.

Complex adaptive systems theory is mostly focused on creating a theoretical understanding of systems that cannot be understood by just looking at individual elements but need to be understood by looking at them holistically (Theise & D’Inverno 2004:17). It is thought that by looking at systems holistically, it is possible to formulate contextually relevant and sustainable solutions to complex problems (Zhou, Wan & Jia 2010). Interestingly, despite its name, complex adaptive system theory does not argue that complex systems and the problems that emerge within them necessarily require equally complex solutions. On the contrary, it is argued that complex systems such as the environment, economy and government often function more effectively if there are only a number of simple rules in place that allow for orderly and self-organising behaviour to emerge (Rowe & Hogarth 2005:398). A self-organising system could be exemplified by the example of the functioning of a traffic circle, where there is a simple rule in place governing who has right of way in the circle, but for the most part, drivers entering into the circle guide their decision to enter the circle in response to the actions of other drivers entering and exiting the circle. Self-organising behaviour is seen as essential to help a system and individual in a system to find novel ways to cope with chaotic situations and function at the ‘edge of chaos’. According to Galatzer-Levy (2016:419), complexity, change, development, creativity and adaptive behaviour only really become possible when a system functions at the edge of chaos. Consequently, systems that are regulated using a simple set of rules often adapt more efficiently to challenging situations as individual elements in the system are allowed to self-organise and change their behaviours in line with the challenge presented. This is juxtaposed with heavily regulated systems that cannot self-organise and adapt efficiently because of a reliance on top-down control from a single or central entity (Theise & D’Inverno 2004:18). Continuing the example of the traffic circle cited above, self-organising systems such as a traffic circle might appear chaotic in their functioning; however, they often streamline traffic and the flow thereof much more efficiently than more regulated systems such as traffic lights that are more predictable and regulated in their function but slow down traffic flow, which can lead to traffic congestion during certain parts of the day. In the context of international responses to COVID-19, some studies have emerged in recent months comparing the efficacy of hard lockdowns (government regulated and enforces lockdowns) versus ‘soft’ lockdowns (limited government regulation, but an emphasis on individual self-organising behaviour). In one such study, Jamison et al. (2020:9–10) found that allowing for self-initiated behaviour change prior to implementations of restrictive government rules and regulations was almost equally effective in reducing the growth rate of COVID-19 deaths, with the former approach reducing the numbers by 9% and the latter by 14%. Importantly, Jamison (2020:11) and Kabiraj et al. (2021:6) mentioned that although government-imposed hard lockdowns are at best, moderately more effective than soft lockdowns, the socioeconomic disruption brought about by their prolonged implementation would likely not be a viable option in developing countries in Africa and Asia. In this instance, a country such as Tanzania has effectively been forced to take on more of a soft lockdown approach, to protect its economy that is largely dependent on tourism. Interestingly, Haider et al. (2020:9) highlighted that Tanzania with its soft lockdown approach based largely on positive self-organising behaviours (social distancing, mask wearing, and increased hand cleaning) by its citizens has had substantially less COVID-19 cases and deaths, than South Africa, which has one of the strictest lockdowns in Africa.

The question can now be asked, what are the possible implications of simple rules and self-organising behaviour for how the South African government has responded to the COVID-19 outbreak in the country? As mentioned above, systems that function optimally are often those that are not overly regulated and with only simple rules, which allow individuals or individual entities in the system to regulate themselves in such a way that novel and efficient coping mechanism can emerge. Now, the question could be asked, *what would constitute a simple rule in the case of managing a pandemic disease outbreak*? Arguably, the most effective way for a communicable disease such as COVID-19 to spread is through the mass gathering of persons in close proximity to each other (Wilder-Smith & Freedman 2020:2). Thus, a simple rule in the context of addressing COVID-19 would be any rule that effectively limits amounts of persons in any one place at one time and the distance between persons in a certain space. The mandatory wearing of face masks and other PPE would also constitute a simple rule (Cuevas 2020:3; Feng et al. 2020:435).

**FIGURE 1:**
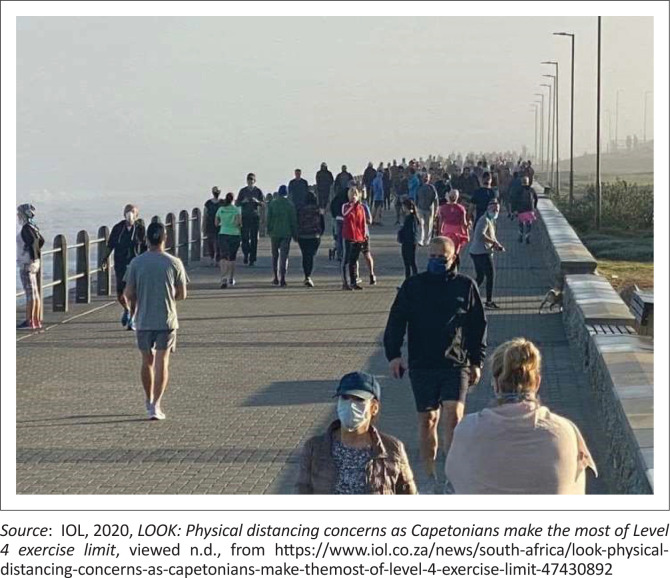
Exercise on Sea Point Promenade, Cape Town, during lockdown level 4.

Although all these rules have been put in place at various times of South Africa’s COVID-19 response, the addition of additional rules and regulations has in some instance led to an inefficient and contradictory response to emerge. A prime example of this is illustrated in Figure 1 that shows citizens of Cape Town exercising at the start of lockdown level 4. In this case, during the first period of lockdown and later lockdown level 5, citizens were prohibited from engaging in running, walking and cycling in public because of government’s belief that these activities could aid the spread of the disease (a notion that was not backed up by scientific evidence). Once lockdown level 4 was announced, government decided to allow citizens to exercise, but this right to exercise was subject to regulations including only being allowed to exercise between 6 am and 9 am and only being allowed to exercise with a 5 km radius of one’s home (Haider et al. 2020:6). Again, no scientific basis was provided for both these regulations. However, the consequence of their implementation is clear in the given figure, that is, forcing people into the same space and thereby creating a lack of social distancing and a favourable environment for the spread of COVID-19. Arguably, in normal circumstances, people would spread their exercise throughout the day, over different distances and spaces within and around the city, leading to a limited number of persons in any one space at any given time. Thus, allowing individuals to continue their regular exercise routine or allowing them to self-organise, except for the addition of simple social distancing regulations, for example, not running in groups or clubs and mandatory mask wearing, could have allowed for a more efficient response to COVID-19 transmission to emerge, than that emerged because of government over regulation.

By their very nature, complex systems and the complex problems that emerge within them resist being controlled through centralised control mechanisms (such as Government policy) as there are too many individual and contextual factors at play influencing the systems behaviour (Levin 1998:432). Government could never hope to control the behaviour of all individual and contextual factors that influence the system and should leave the socioeconomic system to self-organise and create self-regulatory behaviour amongst the individual elements in the system as it will allow for a more streamlined and context-specific adaptation to occur for COVID-19. This notion seems to be supported by Jamison (2020:11) who found that overly stringent regulations associated with hard lockdowns, that is, closing of schools and imposing stay at home rules, can be associated with the ban of exercise during most of the South Africa’s lockdown level 4. It had statistically insignificant effects in reducing the spread and death rates associated with COVID-19. In addition, Haider et al. (2020:8) argued that in his study of nine sub-Saharan African countries, hard lockdown regulations such as curfews and stay at home rules have actually had counterproductive impacts in combating the spread of COVID-19, because of the large numbers of densely populated informal settlements in the region.

As any proposed approach to respond to COVID-19, such as self-organising behaviour and simple rules might also have its relative weaknesses, for instance, if the community observes any reduction in COVID-19 infection cases, it might create the impression that the risk of infection is very low and they would adjust (self-organise) their behaviour, by being less vigilant with mask wearing or social distancing behaviour or even going as far as to completely circumvent all established COVID-19 protocols. However, as Herby (2021:4–5) highlighted, these issues are not exclusive to a soft lockdown approach characterised by self-organisation and that most countries following hard lockdown approaches have faced similar problems, in addition to the tremendous socioeconomic and psychological consequences brought on by hard lockdowns in the long run. In the experience of both Sweden and South Korea, the use of soft lockdown approaches governed by simple rules allowed citizens to self-organise, which it freed up government to focus on extensive testing, tracing and information campaigns, all of which have been shown to play a major role in effectively reducing the spread of COVID-19 (Moon 2020:654; Pierre 2020:480). It is important to note that highlighting South African government’s response to COVID-19 could be guided by concepts such as simple rules and self-organising behaviour, which is not saying that government should become totally cavalier in its response, rather it is a recognition that alternative approaches of responding to COVID-19 have emerged globally that could provide an alternative approach for the South African government to deal with this and future disease outbreaks of this scale.
